# Involvement of metformin and AMPK in the radioresponse and prognosis of luminal versus basal-like breast cancer treated with radiotherapy

**DOI:** 10.18632/oncotarget.2683

**Published:** 2014-11-04

**Authors:** Yimin Zhang, Sarah J. Storr, Kerstie Johnson, Andrew R. Green, Emad A. Rakha, Ian O. Ellis, David A.L. Morgan, Stewart G. Martin

**Affiliations:** ^1^ Academic Unit of Clinical Oncology, Division of Cancer and Stem Cells, School of Medicine, University of Nottingham, Nottingham University Hospitals NHS Trust, City Hospital Campus, Nottingham, UK; ^2^ Clinical Oncology, Nottingham University Hospitals NHS Trust, City Hospital Campus, Nottingham, UK; ^3^ Histopathology, Division of Cancer and Stem Cells, School of Medicine, University of Nottingham, Nottingham University Hospitals NHS Trust, City Hospital Campus, Nottingham, UK

**Keywords:** metformin, AMPK, radiosensitivity, breast cancer, luminal phenotype

## Abstract

Metformin is under evaluation as a potential anticancer agent. Expression of total and phospho(Thr172)-adenosine monophosphate-activated kinase-α (AMPKα and pAMPKα(Thr172) respectively), a main metformin target, was examined in radiotherapy treated breast cancers and metformin's ability to modulate Trx system expression and breast cancer radiosensitivity evaluated in vitro.

AMPKα and pAMPKα(Thr172) expression was assessed using a discovery (n=166) and validation cohort (n=609). Metformin's role in regulating radioresponse, and Trx family expression, was examined via clonogenic assays and Western blots. Intracellular reactive oxygen species (ROS) levels, cell cycle progression and apoptosis were assessed by flow cytometry.

High AMPKα expression associated with improved local recurrence-free (*P*=0.019), relapse-free (*P*=0.016) and breast cancer-specific survival (*P*=0.000065) and was, from multivariate analysis, an independent prognostic factor from the discovery cohort. From the validation cases AMPKα expression associated with relapse-free and breast cancer-specific survival in luminal breast cancers. Metformin substantially increased radiosensitivity, intracellular ROS levels and reduced Trx expression, in luminal breast cancer cells, but had little effect on basal phenotype cells.

In conclusion, high AMPKα expression associates with improved prognosis, especially in luminal breast cancer. Metformin preferentially radiosensitises luminal breast cancer cells, potentially due to alterations to intracellular ROS levels via modulation of Trx family protein expression.

## INTRODUCTION

Metformin (1,1-dimethylbiguanide hydrochloride) is a biguanide used worldwide to treat type II diabetes and pre-diabetic syndromes [1]. The physiological mechanism of its action in diabetes is to enhance glucose utilization, increase insulin sensitivity and reduce hepatic glucose production and free fatty acid utilization [2]. Its primary effect is based on interfering with respiratory complex I, reducing ATP production [3], and leading to the activation of adenosine monophosphate-activated kinase (AMPK) [4, 5]. Diabetic patients treated with metformin, but not other anti-diabetic drugs, have reduced incidence and better survival from cancer of many organs, including colorectal, liver, pancreatic, rectal, breast, prostate cancer and upper-tract urothelial carcinoma [6-12]. However, discordance exists in prostate cancer as some retrospective studies have reported that metformin use was not associated with risk reduction in systemic progression and all-cause mortality in patients treated with radical prostatectomy [13-16].

AMPK, a serine/threonine kinase, functions as an energy sensor and metabolic master switch, and is activated under conditions of increasing cellular AMP:ATP ratios, such as hypoxia, heat shock and ischemia [17]. AMPK is a heterotrimeric protein composed of a catalytic α subunit (63 kDa) and regulatory β (40 kDa) and γ (38 kDa) subunits. Each is expressed as functionally redundant isoforms including α1, α2, β1, β2, γ1, γ2 and γ3, giving 12 different possible combinations of holoenzyme. Upon activation, the α subunit is phosphorylated at the Thr172 residue (reviewed in [18]). Activated AMPK elevates cellular energy levels by stimulating energy producing catabolic pathways and inhibiting anabolic energy consuming pathways. AMPK can be activated by up-stream proteins including liver kinaseB1 (LKB1) [19] and ataxia teleangiectasia mutated (ATM) kinase [20]. The activation of AMPK inactivates mammalian target of rapamycin (mTOR), a stimulator of cancer cell growth and proliferation frequently hyper-activated by genetic alterations in cancer [21]. The anti-tumour effects of metformin have been shown to be dependent on such AMPK activation [22-25], however antineoplastic effects may also be independent of AMPK activation with, for example, altered NF-kB signalling being implicated [26].

Metformin and AMPK have recently been shown to be involved in regulating the radioresponse of cancer cells. Metformin radiosensitised FSaII mouse fibrosarcoma cells and human breast cancer MCF7 [27], lung cancer cells A549 and H1299 [28, 29] and preferentially killed cancer stem cells, by activating AMPK and suppressing mTOR [27]. AMPK inhibition induced radioresistance of lung cancer cells A549 and H1299 in normal culture conditions [28], with glucose starvation reversing this [30]. AMPK is phosphorylated by irradiation in an ATM dependent manner [30], but independent of LKB1 [28]. Metformin is reported to be involved in redox regulation; reducing intracellular reactive oxygen species (ROS) levels in primary human aortic endothelial cells by upregulating expression of thioredoxin (Trx) *via* the AMPK-FOXO3 pathway [31]. It has also been shown to inhibit thioredoxin-interacting protein (Txnip) mRNA as well as protein expression in HeLa cells [32].

The Trx system is a central enzyme family that regulates intracellular redox homeostasis and plays an important role in regulating the effects of irradiation on cancer cells [33]. Trx is a central part of the Trx system that also includes thioredoxin reductase (TrxR) and Txnip [34]. Trx is reduced, into its biologically active form, by TrxR in a NADPH-dependent manner and in turn reduces oxidized cysteine groups on down-stream proteins [35]. Txnip is the negative regulator of Trx, which directly interacts with the catalytic active centre to block the reducing activity of Trx as well as the interaction between Trx and its down-stream factors [36].

The aims of this study were to determine the expression, and clinical importance, of total- and phospho(Thr172)- AMPKα in early-stage invasive breast cancer from patients treated with radiotherapy and to investigate the effect of metformin on the radiosensitivity of different phenotypes of breast cancer cells, assessing if changes in redox homeostasis, due to alterations in Trx system proteins, played a role in any altered radiosensitivity.

## RESULTS

### AMPKα and pAMPKα(Thr172) staining location and frequency – in the discovery cohort

**Figure 1:**
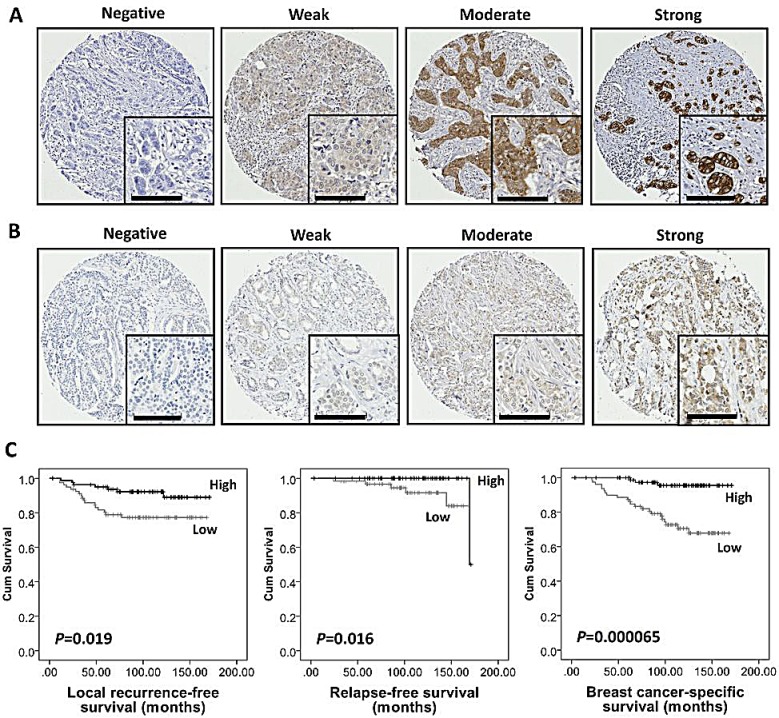
High AMPKα expression was associated with good prognosis in breast cancer in the discovery cohort Representative photomicrographs of negative, weak, moderate and strong staining of (A) AMPKα and (B) pAMPKα(Thr172). Photomicrographs are at ×10 magnification with ×20 magnification inset box where scale bar shows 100 μm. (C) Kaplan-Meier analysis of local recurrence-free, relapse-free and breast cancer-specific survival showing the impact of AMPKα expression in the discovery cohort of 166 patients with significance determined using the log-rank test. High AMPKα expression is associated with lower local recurrence risk (*P*=0.019), better relapse-free survival (*P*=0.016) and breast cancer-specific survival (*P*=0.000065).

Both pAMPKα(Thr172) and AMPKα demonstrated a mixture of diffuse and granular cytoplasmic staining. Heterogeneous staining was shown between, as well as within, certain tumour cores for both markers, varying from weak to intense staining. Cytoplasmic staining of both markers was scored: pAMPKα(Thr172) had a median H-score of 98, ranging between 0 and 200; and AMPKα had a median H-score of 93, ranging between 0 and 228. Figure 1A and B illustrates the staining pattern for both markers. There was a marginal positive correlation between both markers (r=0.305, *P*=0.000084). X-tile bioinformatics software was used to obtain an unbiased optimal H-score cut-point for each protein based on patient outcome, and dichotomised H-scores into low and high scores. The cut-point for pAMPKα(Thr172) was 65 with 30% (48 of 162) of cases having a low score; and the cut-point for AMPKα was 90 with 49% (80 of 163) of cases having a low score. A small number of tissue microarray (TMA) cores were not assessed due to missing cores or insufficient representative tumour within a core.

### Relationship of AMPKα and pAMPKα(Thr172) expression with clinicopathological variables and clinical outcome – in the discovery cohort

The expression of AMPKα and pAMPKα(Thr172) was assessed for association with clinicopathological variables in the discovery cohort (Table 1). High AMPKα expression was significantly associated with smaller tumour size (χ^2^=3.97, d.f.=1, *P*=0.046), low grade (χ^2^=28.338, d.f.=2, *P*=0.000001), low Nottingham Prognostic Index (NPI) score (χ^2^=18.84, d.f.=2, *P*=0.000081) and estrogen receptor (ER) positive status (χ^2^=38.69, d.f.=1, *P*<0.000001). The expression of pAMPKα(Thr172) was not significantly associated with any of the clinicopathological variables.

**Table 1:** Association between AMPKα protein expression and clinicopathological variables

Variable	Discovery cohort AMPKα (n=163)			Validation cohort AMPKα (n=479)		
	Low	High	*P*-value	Low	High	*P*-value
Age (years)						
≤40	3	4	1	22	22	**0.017**
>40	77	79		140	295	
Size (cm)						
≤2	63	73	**0.046**	121	257	0.105
>2	17	8		41	60	
Stage						
I	65	63	0.763	117	235	0.904
II	13	17		38	69	
III	2	2		7	13	
Grade						
I	6	25	**0.000001**	9	59	**<0.000001**
II	24	37		42	139	
III	50	19		111	119	
Node status						
Negative	64	62	0.595	112	220	0.982
Positive	16	19		39	77	
NPI						
Good (<3.4)	22	47	**0.000081**	34	143	**0.000001**
Intermediate (3.4-5.4)	55	28		109	153	
Poor (>5.4)	3	6		19	21	
Vascular invasion						
Negative	60	65	0.518	113	200	0.109
Positive	20	17		46	114	
ER						
Negative	33	1	**<0.000001**	70	45	**<0.000001**
Positive	47	80		90	261	
PgR						
Negative	ND			90	87	**<0.000001**
Positive				62	207	
HER2						
Negative	ND			135	277	0.481
Positive				21	35	
Basal phenotype						
Non-basal	ND			101	254	**0.000003**
Basal				52	45	
Classification						
Luminal	ND			91	265	**<0.000001**
Triple negative				55	28	
HER2+				12	11	

The P-values are resultant from the Pearson χ^2^ test of association. Significant P-values are indicated in bold. The discovery and validation cohorts were comprised of 166 and 609 patients respectively; however, scores were not available for every patient (163 of 166 in the discovery cohort and 479 of 609 in the validation cohort). The number of observations for each cohort is shown for each clinicopathological variable; the table does not include the number of observations where clinicopathological data were not available. Abbreviations: ND, not determined.

Kaplan-Meier analysis showed that high AMPKα expression was associated with better outcome in terms of lower local recurrence risk (*P*=0.019), longer relapse-free survival (*P*=0.016) and breast cancer-specific survival (*P*=0.000065) (Figure 1C), while pAMPKα(Thr172) was not associated with breast cancer outcome in this cohort. Multivariate Cox regression analysis including tumour size, stage (i.e. TNM stage), grade, node status, NPI, vascular invasion and ER status (with individual Kaplan-Meier statistics of *P*=0.048, *P*=0.000079, *P*=0.000025, *P*=0.019, *P*=0.000482, *P*=0.003 and *P*=0.007, respectively), showed that AMPKα expression was independently associated with breast cancer-specific survival (Hazard Ratio (HR) = 0.16; 95% confidence interval (CI) = 0.04-0.63; *P*=0.009); and such analysis, including tumour stage (with individual Kaplan-Meier statistics of *P*=0.001), showed that AMPKα expression was also independently associated with relapse-free survival (HR = 0.36; 95% CI = 0.15-0.87; *P*=0.023) (Table 2). AMPKα expression was not, however, independently associated with local recurrence in multivariate analysis that included ER status and tumour size (HR = 0.52; 95% CI = 0.19-1.43; *P*=0.206).

**Table 2:** Multivariate Cox Regression analysis of factors associated with breast cancer-specific survival and relapse-free survival for the discovery cohort

	Breast cancer-specific survival			Relapse-free survival		
Variable	HR	95% CI	*P*-value	HR	95% CI	*P*-value
AMPKα expression	0.16	0.04 to 0.63	**0.009**	0.36	0.15 to 0.87	**0.023**
Stage	2.24	0.54 to 9.37	0.269	1.17	0.51 to 2.69	0.708
Grade	2.61	0.68 to 10.08	0.164	N/A		
Size (≤ / > 2 cm)	1.48	0.49 to 4.45	0.488	N/A		
Node status (−/+)	0.84	0.09 to 7.72	0.880	N/A		
NPI	1.21	0.24 to 6.17	0.821	N/A		
Vascular invasion	2.19	0.91 to 5.29	0.081	N/A		
ER (−/+)	0.93	0.37 to 2.37	0.882	N/A		

Abbreviation: HR, Hazard Ratio; CI, confidence interval; N/A, not applicable. Significant P-values are indicated by bold.

### Relationship of AMPKα expression with clinicopathological variables and clinical outcome – in the validation cohort

To verify the finding that AMPKα expression was associated with prognosis in early-stage invasive breast cancer patients treated by breast-conserving surgery plus radiotherapy, an independent patient validation cohort was investigated. The staining pattern of AMPKα was similar to that of the discovery cohort. The median H-score was 145 and ranged between 0 and 300. The X-tile determined cut-point was 115 with 34% (162 of 479) of cases having a low score. Similar to the discovery cohort, high AMPKα expression was significantly associated with features of good prognosis including low grade (χ^2^=43.42, d.f.=2, *P*<0.000001), low NPI score (χ^2^=27.32, d.f.=2, *P*=0.000001) and ER positive status (χ^2^=47.68, d.f.=1, *P*<0.000001). High AMPKα expression was also associated with some of the additional biomarkers available for this cohort, including progesterone receptor (PgR) positive status (χ^2^=36.72, d.f.=1, *P*<0.000001), and basal-phenotype negative status (χ^2^=21.53, d.f.=1, *P*=0.000003). High AMPKα expression was significantly associated with older patients (χ^2^=5.67, d.f.=1, *P*=0.017), but not with tumour size (Table 1).

**Figure 2:**
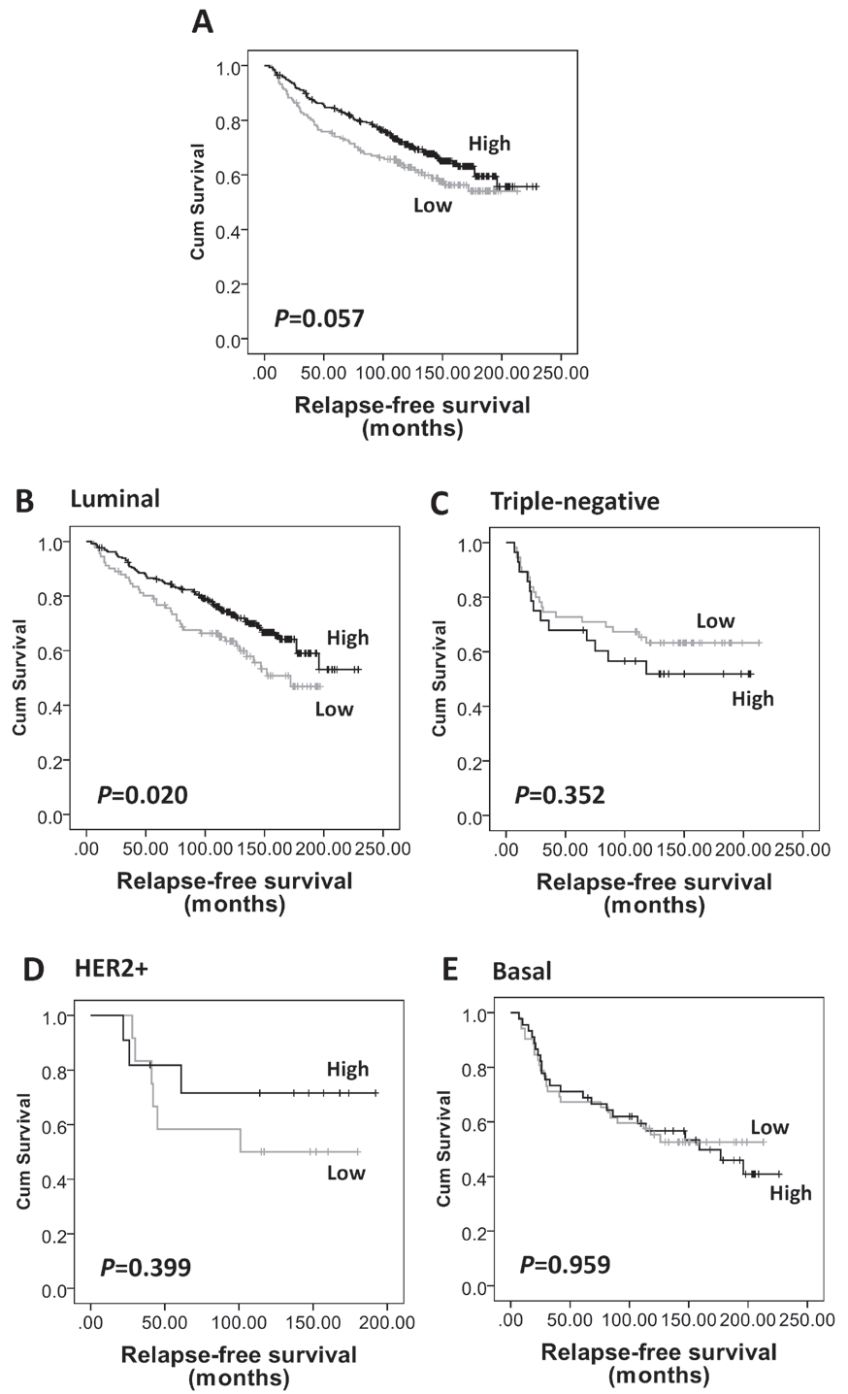
Kaplan-Meier analysis of relapse-free survival showing the impact of AMPKα expression in the validation cohort of 609 patients with significance determined using the log-rank test (A) AMPKα expression is not associated with relapse-free survival in the whole cohort (*P*=0.057). (B) High AMPKα expression is associated with a low risk of disease relapse in luminal breast cancer subgroup (n=453, *P*=0.020), but not in (C) triple-negative (n=108, *P*=0.352), (D) HER2+ (n=26, *P*=0.399), and (E) basal-like (n=117, *P*=0.959) breast cancer subgroups.

**Figure 3:**
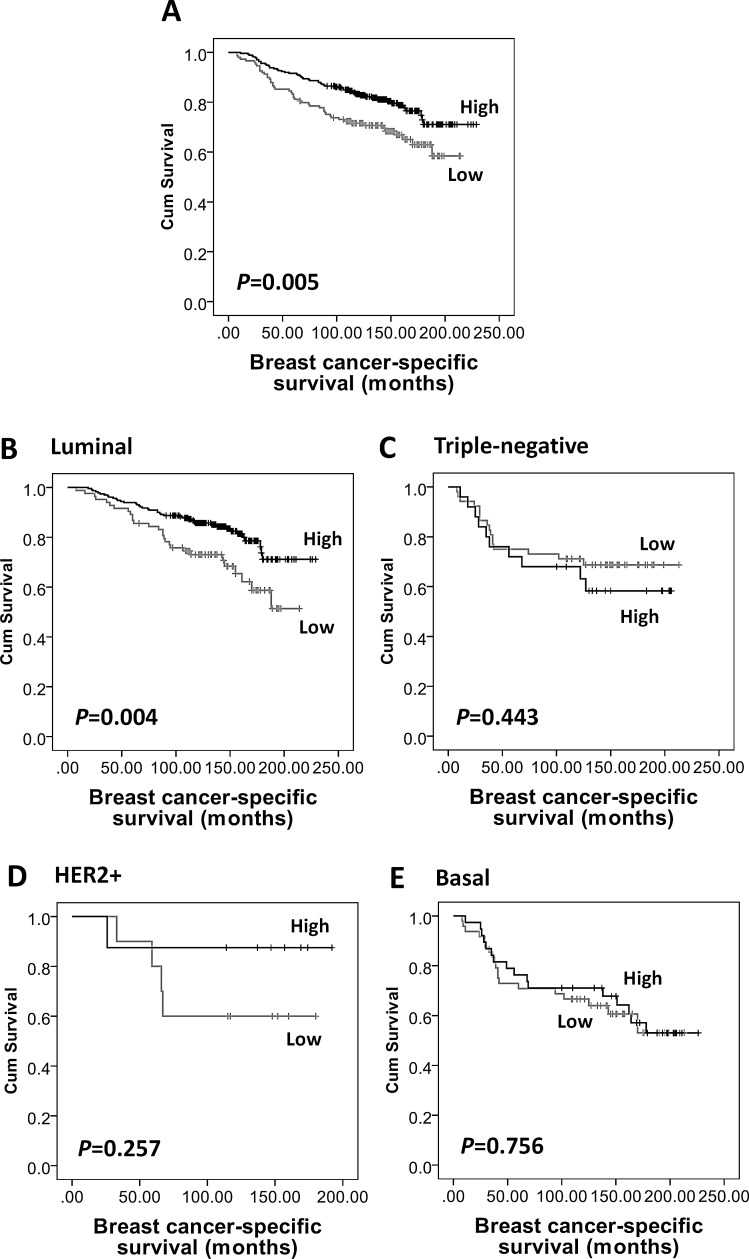
Kaplan-Meier analysis of breast cancer-specific survival showing the impact of AMPKα expression in the validation cohort of 609 patients with significance determined using the log-rank test (A) High AMPKα expression is associated with a low risk of breast specific death in the whole cohort (*P*=0.005) and in (B) luminal breast cancer subgroup (n=453, *P*=0.004), but not in (C) triple-negative (n=108, *P*=0.443), (D) HER2+ (n=26, *P*=0.257), or (E) basal-like (n=117, *P*=0.756) breast cancer subgroups.

Kaplan-Meier survival analysis showed that high AMPKα expression was associated with longer breast cancer-specific survival (*P*=0.005, Figure 3A), but not with local recurrence-free (*P*=0.328) or relapse-free survival (*P*=0.057, Figure 2A). In multivariate Cox regression analysis, including tumour size, stage, grade, node status, NPI, vascular invasion, ER, PgR, human epidermal growth factor receptor 2 (HER2) and basal-phenotype status (with individual Kaplan-Meier statistics of *P*=0.041, *P*<0.000001, *P*=0.00002, *P*=0.000005, *P*<0.000001, *P*=0.002, *P*=0.004, *P*=0.000428, *P*=0.002 and *P*=0.000073, respectively), AMPKα expression was not independently associated with breast cancer-specific survival in all breast cancer phenotypes (HR = 0.66; 95% CI = 0.41-1.07; *P*=0.089) but significant results were obtained with the luminal-phenotype group separately and are described below.

### Relationship of AMPKα expression with clinical outcome of luminal-phenotype breast cancer – in the validation cohort

As AMPKα expression was significantly associated with ER, PgR and basal-phenotype status, we further investigated the prognostic significance of AMPKα expression in different subtypes of breast cancer in the validation cohort. As shown in Supplementary Table S1, of the 609 patients, 77% (n=453) tumours were of luminal-phenotype, 18% were triple-negative (n=108), 4% (n=26) were HER2+ and 21% (n=117) were basal-like. High AMPKα expression was associated with longer relapse-free survival (*P*=0.02, Figure 2B) and breast cancer-specific survival (*P*=0.004, Figure 3B) in luminal-phenotype tumours. No similar associations were identified in the HER2+, basal-like or triple-negative classes. Multivariate Cox regression analysis demonstrated that AMPKα expression was independently associated with relapse-free survival (HR = 0.57; 95% CI = 0.38-0.86; *P*=0.008; potential confounding factors: stage, node status, NPI, vascular invasion and HER2 with individual Kaplan-Meier statistics of *P*=0.001, *P*=0.015, *P*=0.000282, *P*=0.004 and *P*=0.001, respectively) and breast cancer-specific survival (HR = 0.48; 95% CI = 0.28-0.82; *P*=0.007; potential confounding factors: stage, grade, node status, NPI, vascular invasion, PgR and HER2 with individual Kaplan-Meier statistics of *P*<0.000001, *P*=0.000331, *P*=0.000001, *P*<0.000001, *P*=0.002, *P*=0.01 and *P*=0.000005, respectively) in luminal-phenotype breast cancer cases when potential confounding factors were included (Table 3).

**Table 3:** Multivariate Cox Regression analysis of factors associated with breast cancer-specific survival and relapse-free survival for luminal phenotype disease in the validation cohort

	Breast cancer-specific survival			Relapse-free survival		
Variable	HR	95% CI	*P*-value	HR	95% CI	*P*-value
AMPKα expression	0.48	0.28 to 0.82	**0.007**	0.57	0.38 to 0.86	**0.008**
Stage	3.60	1.75 to 7.42	**0.001**	2.62	1.38 to 4.97	**0.003**
Grade	1.71	0.88 to 3.33	0.114	N/A		
Node status (−/+)	1.07	0.48 to 2.42	0.863	0.66	0.29 to 1.48	0.310
NPI	0.82	0.36 to 1.86	0.633	0.85	0.57 to 1.28	0.435
Vascular invasion	1.53	0.90 to 2.63	0.119	1.68	1.12 to 2.51	**0.011**
PgR (−/+)	0.51	0.29 to 0.90	**0.020**	N/A		
HER2 (−/+)	2.59	1.28 to 5.25	**0.008**	2.12	1.22 to 3.70	**0.008**

Abbreviation: HR, Hazard Ratio; CI, confidence interval; N/A, not applicable. Significant P-values are indicated by bold.

### Metformin sensitises luminal breast cancer cells to irradiation but not basal phenotype

**Figure 4:**
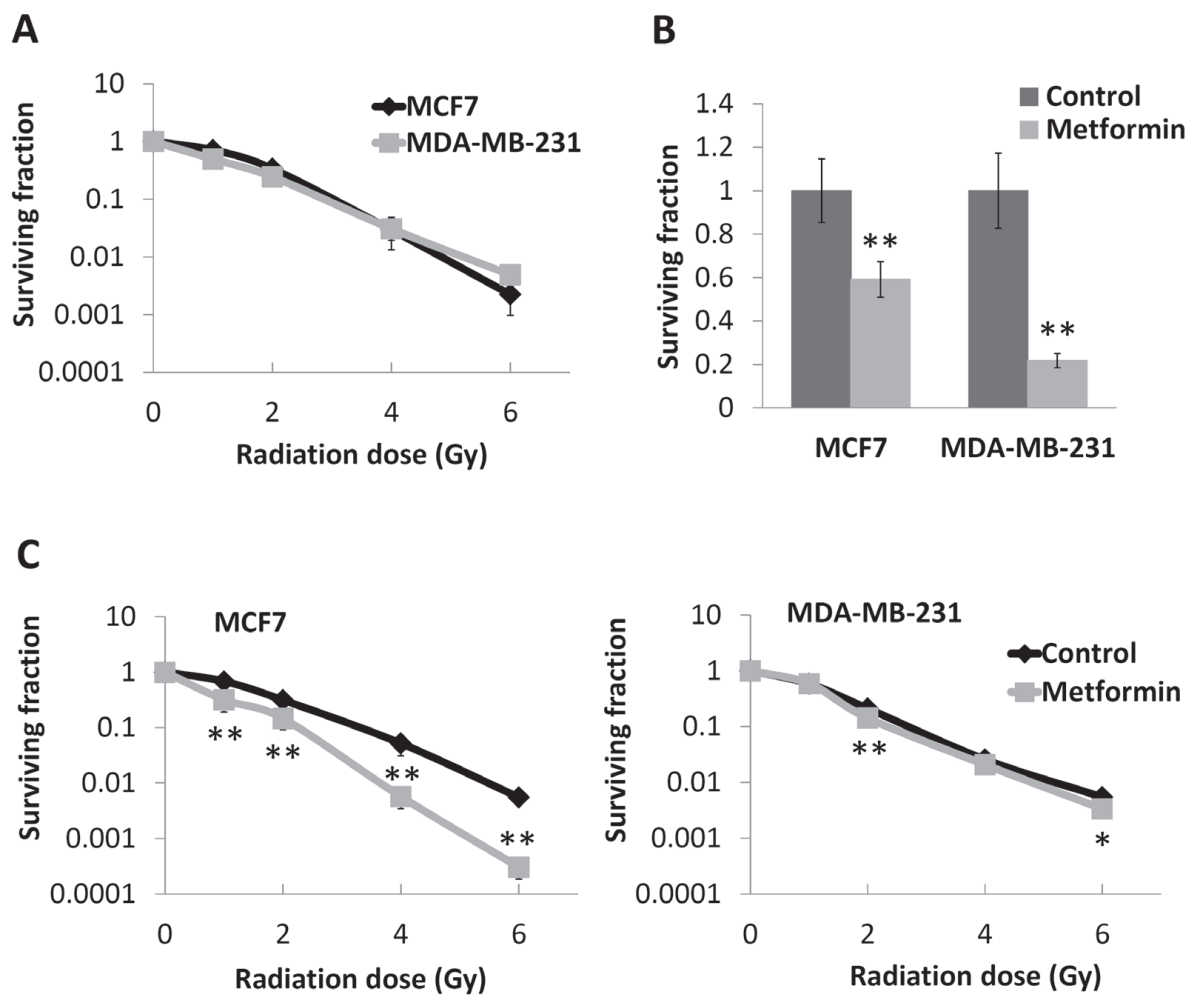
Effects of metformin on survival and radioresponse of breast cancer cell lines (A) Luminal MCF7 and basal-like MDA-MB-231 breast cancer cells were subjected to single dose irradiation and compared with sham irradiated cells (0 Gy) as control. Clonogenic assay was performed to assess the surviving fraction of cells. (B) Both cell lines were treated with 10 mM of metformin for 48 hours (cells without metformin treatment as control), and clonogenic assays were performed to assess the surviving cells. Values were plotted as percentage of control. (C) MCF7 and MDA-MB-231 cells after metformin treatment were subjected to single dose irradiation, and clonogenic assay was used to assess the surviving cells. PEs for MDA-MB-231 and MCF7 cells in clonogenic assay were 52.83% (± 7.43%) and 30.33% (± 6.30%), respectively. Data represent the mean ± SD of three independent experiments, with each experiment containing six parallel data sets. *P<0.05, **P<0.01 *vs.* control.

As radiotherapy is a crucial component of breast-conserving therapy, and to take such IHC based expression studies forward, we assessed whether metformin, a known modulator of AMPK activity could affect the radiosensitivity of different breast cancer phenotypes. Both MCF7 and MDA-MB-231 cells exhibited similar radiosensitivities under control conditions (Figure 4A). The IC50 for metformin in both cell lines, determined from cell growth curve experiments (data not shown), was 10 mM. This drug concentration was used for radiosensitivity studies and was initially assessed for drug alone cytotoxicity using clonogenic survival. As shown in Figure 4B, metformin exhibited significant cytotoxicity to both cell lines; with an increased level of cytotoxicity observed with the basal-like breast cancer MDA-MB-231 cells (surviving fraction (SF)=21.75% of control, *P*<0.01) compared to the luminal MCF7 cells (SF=59.14% of control, *P*<0.01). Interestingly, the radiosensitisation effect of metformin did not parallel its cytotoxic effect. Metformin caused little change in the radiosensitivity of MDA-MB-231 cells but a substantial increase in radiosensitivity of MCF7 cells (sensitiser enhancement ratio (SER) =1.5 at 1% SF) (Figure 4C). The SF at 6 Gray (Gy) for MCF7 cells treated with metformin in combination was 0.03% but 0.55% for radiation alone (*P*<0.01, Figure 4C).

### Metformin elevated intracellular ROS production in luminal breast cancer cells but not basal phenotype

**Figure 5:**
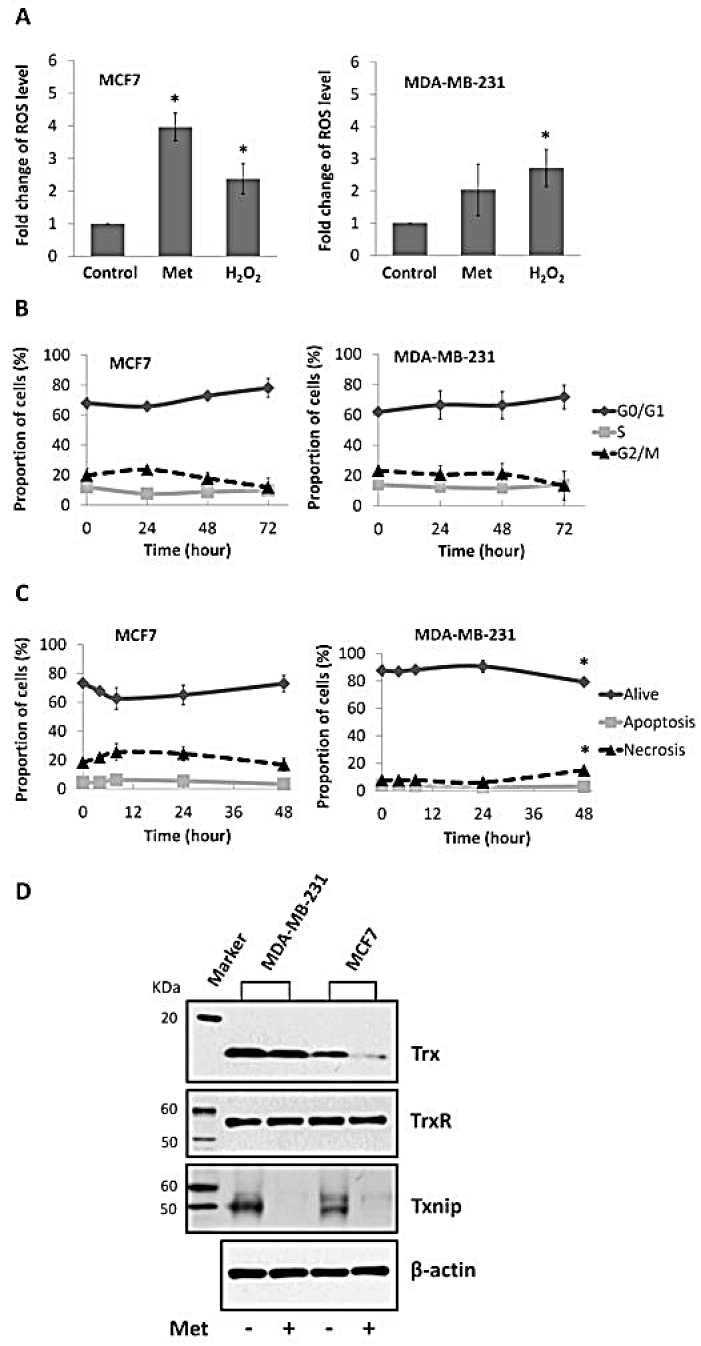
Effects of metformin on ROS level, cell cycle, apoptosis, and expression of Trx system proteins in breast cancer cell lines (A) Flow cytometry analysis of intracellular ROS level of MCF7 and MDA-MB-231 breast cancer cells treated with 10 mM metformin (Met) for 48 hours, positive control (H_2_O_2_) and negative control cells. MFIs were calculated and plotted as fold change of control. Cells treated with 10 mM metformin for different period of time as indicated (72 hours means that cells were treated with metformin for 48 hours and spared for extra 24 hours) were subject to flow cytometry analysis of (B) cell cycle and (C) apoptosis. Percentages of cells were calculated and presented as mean ± SD of three independent experiments. *P<0.05 *vs.* control. (D) Cells were treated with 10 mM metformin for 48 hours (cells without treatment as control). Western blot was performed to assess the expression of Trx (MW=12KDa), TrxR (MW=55KDa) and Txnip (MW=50KDa) in cells, with β-actin (MW=42KDa) as internal control. Experiments were repeated three times and the representative blots are presented.

To explore the reason for the differential radiosensitising effects of metformin on breast cancer cells, intracellular ROS levels were assessed by flow cytometry. As shown in Figure 5A H_2_O_2_ induced ROS to a similar level in both lines but after metformin treatment, intracellular ROS levels were elevated to 4- fold of control in MCF7 cells (*P*<0.05), with no significant alterations in MDA-MB-231 cells (*P*>0.05).

As radiosensitivity can be influenced by the mode of cell death and by perturbations in cell cycle distribution, flow cytometry assessments of apoptosis and the cell cycle were conducted. As shown in Figure 5B and 5C, metformin had no effect on either cell cycle or apoptosis of MCF7 cells. In MDA-MB-231 cells, metformin induced a slight increase in the percentage of necrotic cells (1.94 -fold of control, *P*<0.05, Figure 5C), however it did not affect the apoptotic or cell cycle response of this line.

### Metformin differentially regulates expression of Trx family proteins in breast cancer cells

Western blot analysis of Trx, TrxR and Txnip was conducted to assess whether any altered ROS homeostasis by metformin might involve the Trx system. The endogenous expression levels of Trx and Txnip were lower in MCF7 than in MDA-MB-231, whilst TrxR expression was similar. Metformin decreased Trx expression levels in MCF7 but had no effect on MDA-MB-231 cells. In both cell lines, expression of Txnip was dramatically attenuated by metformin treatment. TrxR expression was not affected by metformin treatment in either cell line (Figure 5D).

## DISCUSSION

Retrospective studies have found that use of the antidiabetic drug metformin in diabetic patients' results in a reduced incidence of, and better survival from, breast cancer [12, 37-39]. However, in a randomized pre-surgical trial including 200 non-diabetic breast cancer patients, no alteration to tumour proliferation, as assessed by Ki67, was observed [40]. Metformin has been reported to be involved in regulating the radioresponse of a number of different cancer cell types, including breast cancer, in an AMPK-dependent manner [27-29, 41]. In order to assess the possible value of metformin as a sensitiser for radiotherapy of breast cancer and AMPK as a potential prognostic factor, the present study first sought to evaluate the expression of AMPK, the target of metformin, in radiotherapy treated early-stage invasive breast cancer patients. Similar to others [42], the current study did not observe any association between pAMPKα(Thr172) expression and prognosis. However, when total-AMPKα expression was assessed, in two independent patient cohorts, expression was associated with patient prognosis. The reason why total-AMPKα but not pAMPKα(Thr172) associated with prognosis is uncertain, but may be due to the differential phosphorylation patterns that can occur with AMPK: in addition to Thr172 AMPKα is also phosphorylated at Thr258 and Ser485 for AMPKα1 and Ser491 for AMPKα2 [43], expression of pAMPKα at other phosphorylation sites, rather than Thr172, may be important for breast cancer prognosis, and should perhaps be addressed in future work.

Although pAMPKα(Thr172) expression has been previously studied using immunohistochemical approaches, total-AMPKα has rarely been assessed. Small studies in thyroid and breast cancer measured levels between normal and cancerous tissue [44, 45] but the current study is the first of its type to determine associations with clinical outcomes. In both the discovery and validation cohorts, high AMPKα expression was significantly associated with low grade, low NPI score and ER positive status, all of which are indicative of better prognosis. High AMPKα expression was associated with patient age in the validation cohort but not in the discovery cohort. The age distribution was, however, different in these two cohorts: the age of patients in the discovery cohort ranged from 31 to 70 *vs.* 18 to 72 in the validation cohort; and the number of patients aged 40 or less occupied 8% of the whole population in the validation cohort, which is nearly twice of that in the discovery cohort (4.2%). AMPKα expression was associated with two additional clinicopathological variables in the validation cohort: PgR and basal-phenotype status; these clinicopathological variables were not available for the discovery cohort. The association of high AMPKα expression with ER, PgR positive and non basal-like tumours may indicate differential expression of AMPKα in different breast cancer phenotypes and requires further verification.

High AMPKα expression was associated with lower local recurrence risk, better relapse-free and breast cancer-specific survival. In multivariate Cox regression analysis AMPKα significantly associated with relapse-free and breast cancer-specific survival independent of possible confounding factors in the discovery cohort. AMPKα expression was significantly associated with breast cancer-specific survival in the validation cohort. As AMPKα expression was related to breast cancer phenotype, the importance of AMPKα expression in prognosis of different subtypes of breast cancer was assessed in the validation cohort. Interestingly, high AMPKα expression associated with better relapse-free and breast cancer-specific survival and in multivariate Cox regression analysis AMPKα expression was also independently associated with relapse-free and breast cancer-specific survival in luminal phenotype breast cancer. Our study is the first to examine the expression of total-AMPKα in breast cancer tissue and to report on its prognostic significance in radiotherapy treated breast cancer, especially in luminal phenotype disease.

Interestingly, such phenotype preference is also observed in the tumour inhibitory effects of metformin, the activator of AMPK, in breast cancer patients. Retrospective studies have shown that metformin use is associated with improved breast cancer-specific survival of diabetic women with luminal [38] and HER2+ breast cancers [39], but did not significantly impact survival outcomes in diabetic patients with triple-negative breast cancer [46]. As a result, we further assessed the effect of metformin on the radioresponse of different phenotypes of breast cancer *in-vitro*. Luminal breast cancer MCF7 and basal-like breast cancer MDA-MB-231 cells were used in this study, and when treated by single-dose irradiation both lines exhibited a similar response. However, when metformin treatment was combined with irradiation, a substantial increase in radiosensitivity of MCF7 cells was observed, with an SER of 1.5, but with little effect on the radiosensitivity of MDA-MB-231 cells. Such results suggest a possible phenotype related mechanism of action for metformin, which can apparently radiosensitise luminal phenotype cells but has limited effect on basal phenotype breast cancer.

To explore the mechanisms of this interesting phenomenon, alterations to redox homeostasis were assessed by examining the change in ROS levels following metformin treatment. Intracellular ROS production is the major mediator for low-LET radiation-induced cell killing in radiotherapy [47]. Metformin induced a significant increase of ROS levels in luminal breast cancer MCF7 cells but had no effect in basal-like breast cancer MDA-MB-231 cells. The differential effects of metformin on ROS production in luminal *vs.* basal-like breast cancer cells may provide a potential explanation for its phenotype preference in radiosensitisation. Others have reported upon metformin regulating intracellular ROS levels but with contradictory findings: metformin reduced the intracellular ROS level in human aortic endothelial cells [31]; but elevated it in primary human fibroblasts [48], which, together with our results, suggest that the effect of metformin in regulating intracellular ROS production may be largely dependent upon the intrinsic characteristics of the different cell types, with no uniform pattern. Current work suggests that the radiosensitisation effects of metformin were not attributable to it altering cell cycle progression or the level of apoptosis. Others have shown, however, that AMPK deficient cells are radioresistant and that such resistance is linked to cell cycle progression and proliferation [49].

Cells have a number of systems available to regulate intracellular ROS production and maintain redox homeostasis including the Trx system [33], which has been shown to be associated with clinical outcome in radiotherapy treated early stage breast cancer [50]. We performed Western blots to analyse the expression changes of Trx family proteins in metformin treated cells: metformin decreased the Trx expression level in MCF7 cells but had no effect on MDA-MB-231 cells. Interestingly, Hou et al. demonstrated that metformin inhibited intracellular ROS by upregulation of Trx expression *via* the AMPK-FOXO3 pathway in human aortic endothelial cells [31], whilst the current study shows the opposite in breast cancer MCF7 cells, with metformin increasing ROS production by decreasing Trx expression. There are probably many factors responsible for such conflicting results, including the concentration and duration of metformin exposure (i.e. 250 μM for 24 hours in the endothelial study *vs.* 10 mM for 48 hours in the current work), and the particular cell line used; however, both studies show the importance of Trx in metformin regulated ROS production. It is also interesting to note the effect of metformin on Txnip, with metformin totally abrogating its expression in both cell lines, which is consistent with previous reports suggesting that metformin inhibits Txnip expression in human hepatocellular carcinoma HepG2 and cervical cancer HeLa cells [32], as well as rat insulinoma INS-1E beta-cells [51].

In summary, the present study shows that high AMPKα expression is associated with a better prognosis of early-stage invasive breast cancer patients treated by breast-conserving surgery and radiotherapy in two independent cohorts. This result was more apparent in luminal phenotype disease with further *in-vitro* characterisations showing that metformin, the activator of AMPK, induced ROS production in luminal breast cancer cells possibly through altered Trx system expression; with little effect in basal phenotype cells. Although further confirmation is required, both *in-vitro* and *in-vivo*, current results suggest that metformin may be a clinically effective radiosensitiser and that its use should potentially be focused on patients with luminal phenotype breast cancer.

## MATERIALS AND METHODS

### Clinical samples

This study is reported according to REMARK (Reporting Recommendations for Tumour Marker Prognostic Studies) criteria [52]. Ethical approval for the study was granted by Nottingham Research Ethics committee 2 under the title “Development of a molecular genetic classification of breast cancer” (C202313). Two independent cohorts were used; a discovery cohort of 166 cases and a validation cohort of 609 cases. Both cohorts were comprised of primary operable early-stage (stage I-III) invasive breast cancers from patients treated by breast-conserving surgery (wide local excision) and radiotherapy at Nottingham University Hospitals. Information on clinical history and outcome is prospectively maintained and patients were assessed in a standardised manner for clinical history and tumour characteristics (see Supplementary Table S1). Breast cancer-specific survival was defined as the time interval (in months) between the date of primary surgery and death resultant from breast cancer; local recurrence-free survival was defined as the time interval (in months) between the start of primary treatment and date of first histological confirmation of recurrent cancer (invasive or *in-situ*) at any site within the treated breast; relapse-free survival was defined as the time interval (in months) between the start of primary treatment and date of disease relapse.

The 166 cases in the discovery cohort were selected from all of the early-stage primary operable invasive breast cancer patients treated by wide local excision and radiotherapy at Nottingham University Hospitals between 1998 and 2006. The selection scheme was based on the local recurrence cases in a chronologic order, where every local recurrence case was included in the study: when a case with local recurrence was chosen, the immediate following 5 suitable cases without local recurrence were included. The median age at diagnosis of this cohort was 56 years (ranging from 31 to 70) and 78% (130 of 166) of patients had stage I disease. The follow-up time was 171 months (median follow-up time 108 months). Supplementary Table S1 shows the full clinicopathological characteristics of this cohort.

The 609 samples in the validation cohort were chosen from a well-characterised consecutive series of early-stage invasive breast cancer patients treated at Nottingham University Hospitals, between 1986 and 1998. From the initial whole series (n=1802), cases treated by wide local excision plus radiotherapy were selected (n=609). The median age of the validation cohort was 54 years (ranging from 18 to 72) and 74% (449 of 609) of patients had stage I disease. The follow-up time was 247 months (median follow-up time 134 months). Data on a wide range of biomarkers was available (Supplementary Table S1); estrogen receptor (ER), progesterone receptor (PgR), human epidermal growth factor receptor 2 (HER2) and basal phenotype status were available for this cohort and have been described previously [53, 54], with basal phenotype being defined as cytokeratin (CK)-5/6 and/or CK-14 positivity [55]. Current cases were classified into three molecular subgroups: ER and/or PgR positive (regardless of HER2 status) was defined as luminal; ER, PgR and HER2 negative was defined as triple-negative; and ER and PgR negative plus HER2 positive was defined as HER2+ [56].

Patient treatment, tissue microarray (TMA) construction and immunohistochemistry (IHC) are described in Supplementary Materials and Methods.

### Cell culture

Two human breast cancer cell lines: MCF7 (luminal subtype), MDA-MB-231 (basal subtype) were used in this study (both were from American Type Culture Collection). All cell lines were cultured at 37°C in a humidified incubator with 5% CO_2_. MCF7 (passage window 15) were maintained in RPMI1640 (Sigma, Dorset, UK) supplemented with 10% iron supplemented donor calf serum (PAA laboratories, Austria) and 1% penicillin/streptomycin (Sigma). MDA-MB-231 (passage window 15) were maintained in minimal essential medium EAGLE (Sigma) supplemented with 0.1 mM non-essential amino acids solution (Sigma), 2 mM L-glutamine(Sigma), 1% penicillin/streptomycin and 10% iron supplemented donor calf serum.

### Cell irradiation and clonogenic survival assay

Cells were sub-confluent at irradiation. X-ray-irradiation was conducted using an RS225 Xstrahl X-ray cabinet irradiation system (Xstrahl Limited, UK) with a single dose of 1, 2, 4 or 6 Gray (Gy). X-rays were delivered at 195 kV, 10 mA, with a dose rate of 0.87 Gy/min. The cabinet was fitted with a 0.5 mm copper filter and used at a 48.4 cm focus-to-skin distance. Sham irradiated cells were used as controls. Cells were exposed to 10 mM of metformin (BioVision, Milpitas, CA) for 48 hours prior to irradiation. Cells were then trypsinised and plated for clonogenic survival assay with six parallel sets. After 18 days colonies were fixed (50% methanol in 0.9% saline solution for 15 min followed by methanol for another 15 min), stained (0.5% crystal violet) and counted as a survivor if containing more than 50 cells. Surviving fraction (SF) was calculated as: number of colonies / (number of cells plated × plating efficiency), where plating efficiency (PE) was defined as: number of control colonies obtained / number of control cells plated. The sensitizer enhancement ratio (SER) was calculated as the radiation dose yielding 1% SF divided by the radiation dose giving the same survival where cells were treated with metformin.

### Measurement of intracellular ROS levels

Sub-confluent cells were treated with 10 mM metformin for 48 hours or, as a ROS positive control, 1 mM of H_2_O_2_ for 1 hour then further incubated with the membrane permeable dye 2′,7′-dichlorodihydrofluorescein diacetate (H_2_DCF-DA, Sigma) in fresh medium at a final concentration of 1 μM for 30 min, at 37°C. Cells were then collected and intracellular ROS levels assessed by measurement of the fluorescence (excitation 500 and emission 520 nm respectively) using a Beckman Coulter FC500 MCL flow cytometer system (Beckman, USA). Data exported from the flow cytometer were analysed using FlowJo7.6.1 software (Tree Star) to obtain the median fluorescence intensity (MFI) of each group.

### Protein extraction and Western blot

Sub-confluent cells, following incubation with 10 mM metformin for 48 hours, were harvested and re-suspended in RIPA buffer (Sigma) supplemented with protease inhibitor cocktail (Sigma). A Bio-Rad protein assay kit (Bio-Rad Laboratories, USA) was used to determine the protein concentration of each sample. Lysates were separated by SDS-PAGE and transferred onto a nitrocellulose membrane. After blocking with 5% (w/v) milk powder in 0.1% PBS/Tween, the nitrocellulose membrane was incubated with primary antibody at 4°C overnight. The primary antibodies used in this study were: goat anti- human Trx antibody (1:1000 dilution; American Diagnostica, Stamford, CT), mouse anti- human TrxR antibody [19A1] (1:2000 dilution; Abcam, Cambridge, UK) and mouse anti- human Txnip antibody (1:1000 dilution; MBL International Corporation, Woburn, USA). HRP-conjugated β-actin antibody (Invitrogen) was used as internal control. Membranes were developed with Amersham ECL reagent (GE Healthcare, UK).

### Cell cycle analysis

Sub-confluent cells were treated with 10 mM metformin for 24 or 48 hours, or treated with metformin for 48 hours and spared for an extra 24 hours (72 hours), then collected and fixed in 70% ethanol in PBS overnight. Fixed cells were then stained using a PBS solution containing 2.5 μg/ml of propidium iodide and 200 μg/ml of RNase for 30 min at 37°C and analyzed using a Beckman Coulter FC500 MCL flow cytometer system (Beckman, USA).

### Annexin V-FITC apoptosis assay

Sub-confluent cells were treated with 10 mM metformin for 4, 8, 24 or 48 hours. Apoptosis of cells was then assessed by using an annexin V-FITC apoptosis detection kit (Invitrogen) according to the manufacturer's instructions and analyzed using a Beckman Coulter FC500 MCL flow cytometer system (Beckman, USA).

### Statistical Analysis

The relationship between categorised protein expression and clinicopathological variables was assessed using the Pearson Chi Square (χ^2^) test of association or Fishers Exact test if a cell count was less than five. Spearman rank order correlations were performed to test the association between expression of AMPKα and pAMPKα(Thr172). Survival curves were plotted according to the Kaplan-Meier method and significance determined using the log-rank test. Multivariate survival analysis was performed by the Cox proportional hazards regression model and included only those parameters that were significant in univariate analysis. Data from *in-vitro* experiments were expressed as the mean ± standard deviation (SD) and analysed using the Student t-test and ANOVA one-way tests. All differences were deemed statistically significant at the level of *P*<0.05. Statistical analysis was performed using SPSS 21.0 software (IBM, Armonk, NY).

## SUPPLEMENTARY MATERIAL TABLE AND FIGURE


