# Supplementary material for: Involvement of metformin and AMPK in the radioresponse and prognosis of luminal versus basal-like breast cancer treated with radiotherapy
Source: Oncotarget. 2014 Nov 4;5(24):12936–49. doi: 10.18632/oncotarget.2683 (PMC4350336; doi:10.18632/oncotarget.2683)
Supplement: Supplementary file 1 [file oncotarget-05-12936-s001.pdf]

# Involvement of metformin and AMPK in the radioresponse and prognosis of luminal versus basal-like breast cancer treated with radiotherapy

## Supplementary Material

## Patient treatment

Patients were managed under a uniform protocol, where all underwent wide local excision followed by radiotherapy. For the validation cohort cases radiotherapy was given in daily 2 Gray (Gy) fractions, to a total dose of 50-55 Gy over 5 weeks and for the discovery cohort the majority of cases were also given this regimen (n=133) with certain ones, however, coming from radiotherapy trials investigating altered fractionation schedules (n=31). Radiotherapy was delivered to the whole breast. During the treatment period for the validation cohort a ‘‘boost’’ to the tumor bed was not routinely given, however a “16 Gy boost” was given to certain patients in the discovery cohort (n=6). Patients received systemic adjuvant treatment on the basis of Nottingham Prognostic Index (NPI), estrogen receptor (ER) status and menopausal status. Patients with an NPI score <3.4 did not receive adjuvant treatment and patients with an NPI score of 3.4 or more were candidates for chemotherapy if they were ER negative or premenopausal; and hormonal therapy if they were ER positive.

## Tissue microarray (TMA) construction and immunohistochemistry (IHC)

Prior to IHC, the specificity of rabbit anti-AMPKα antibody (Abcam, Cambridge, UK) and rabbit anti-pAMPKα (Thr172) (40H9) antibody (Cell Signaling Technology, Danvers, MA) was confirmed by Western blot. This was used in conjunction with peptide blocking assays, in which, antibody was pre-incubated with recombinant peptide/protein prior to use in immunohistochemistry on breast cancer

specimens to ablate staining (see Supplementary Figure S1). AMPKα and pAMPKα(Thr172) staining was initially optimized on breast tumor composite tissue sections comprised of six stage I breast cancer of grade I to III to assess staining patterns and define optimal staining procedures and then assessments on TMA's were conducted.

Expression of AMPKα and phospho-AMPKα (pAMPKα) was investigated using tissue microarrays (TMAs) of the discovery and the validation cohorts which were prepared using 0.6-mm cores as described previously [1]. The discovery cohort TMA consisted of 3 cores per tumor specimen with the validation cohort TMA comprising one core per tumor specimen.

IHC was performed on 4-μm paraffin-embedded sections that were mounted on Superfrost$^{+}$ microscope slides (Menzel-Glaser, Germany). TMA slides were initially deparaffinised in xylene followed by rehydration in industrial methylated spirit (IMS) using a Leica Autostainer XL Staining System ST5010 (Minnesota, USA). Antigen retrieval was performed using 0.01 M sodium citrate buffer (pH 6) in a microwave, 750 W for 10 min followed by 450 W for 10 min. After antigen retrieval slides were stained using a Novolink Novocastra polymer detection kit (Leica, Germany), according to the manufacturers' instructions, with primary antibodies, rabbit anti-AMPKα antibody (1:100 dilution) or rabbit anti-pAMPKα (Thr172) (40H9) antibody (1:50 dilution). Slides were then dehydrated and fixed in xylene before mounting with DPX mounting medium (Leica, Germany). Breast tumor composite sections were included as positive and negative controls with each run, with the negative control having primary antibody substituted for Leica antibody diluent.

Stained slides were scanned using a Nanozoomer Digital Pathology Scanner (Hamamatsu Photonics), at x20 magnification. Expression of pAMPKα(Thr172) and

AMPKα in tumor cells was manually assessed using semi-quantitative immunohistochemistry scoring (H-score) by two independent assessors blinded to the study end points. Staining intensity was assessed as: negative (0), weak (1), moderate (2), and strong (3). H-scores were calculated by multiplying the percentage of positive tumor cells by the staining intensity giving a score ranging between 0 and 300. The second independent assessor scored 30% of the slides for each protein. The mean of the H-scores of the triplicate cores from the same specimen was calculated to represent the expression of the marker for each case from the discovery cohort. Single measure intraclass correlation coefficients between scores were 0.811 and 0.974 for pAMPKα(Thr172) and AMPKα in the discovery TMA, and 0.836 for AMPKα in the validation TMA, respectively, showing excellent concordance between scorers. The H-scores for each protein of the first scorer were then dichotomised using X-tile software (Yale University), prior to analysis against clinicopathological variables and patient outcome [2].

## References

1. Abd El-Rehim DM, Ball G, Pinder SE, Rakha E, Paish C, Robertson JF, Macmillan D, Blamey RW, Ellis IO. High-throughput protein expression analysis using tissue microarray technology of a large well-characterised series identifies biologically distinct classes of breast cancer confirming recent cDNA expression analyses. Int J Cancer. 2005; 116(3):340-350.
2. Camp RL, Dolled-Filhart M, Rimm DL. X-tile: a new bio-informatics tool for biomarker assessment and outcome-based cut-point optimization. Clin Cancer Res. 2004; 10(21):7252-7259.

**Table S1:** Clinicopathological variables for the discovery cohort of 166 patients and the validation cohort of 609 patients assessed using IHC for total and phospho-AMPKα.

| Variable | Discovery cohort N (%) | Validation cohort N (%) |
|---|---|---|
| Age (years) | | |
| ≤40 | 7 (4.2) | 49 (8.0) |
| >40 | 159 (95.8) | 560 (92.0) |
| Size (cm) | | |
| ≤2 | 138 (84.1) | 478 (78.5) |
| >2 | 26 (15.9) | 131 (21.5) |
| Stage | | |
| I | 130 (78.8) | 449 (73.7) |
| II | 31 (18.8) | 135 (22.2) |
| III | 4 (2.4) | 25 (4.1) |
| Grade | | |
| I | 33 (20.1) | 89 (14.6) |
| II | 62 (37.8) | 237 (38.9) |
| III | 69 (42.1) | 283 (46.5) |
| Node status | | |
| Negative | 128 (78.0) | 415 (73.6) |
| Positive | 36 (22.0) | 149 (26.4) |
| NPI | | |
| Good (<3.4) | 71(43.3) | 231 (37.9) |
| Intermediate (3.4-5.4) | 84 (51.2) | 332 (54.5) |
| Poor (>5.4) | 9 (5.5) | 46 (7.6) |
| Vascular invasion | | |
| Negative | 128 (77.6) | 407 (67.7) |
| Positive | 37 (22.4) | 194 (32.3) |
| ER | | |
| Negative | 34 (20.7) | 145 (24.5) |
| Positive | 130 (79.3) | 448 (75.5) |
| PgR | | |
| Negative | ND | 223 (39.7) |
| Positive | | 339 (60.3) |
| HER2 | | |
| Negative | ND | 521 (88.3) |
| Positive | | 69 (11.7) |
| Basal phenotype | | |
| Non-basal | ND | 449 (79.3) |
| Basal | | 117 (20.7) |

| | | |
|---|---|---|
| Classification | | |
| Luminal | ND | 453 (77.2) |
| Triple negative | | 108 (18.4) |
| HER2+ | | 26 (4.4) |

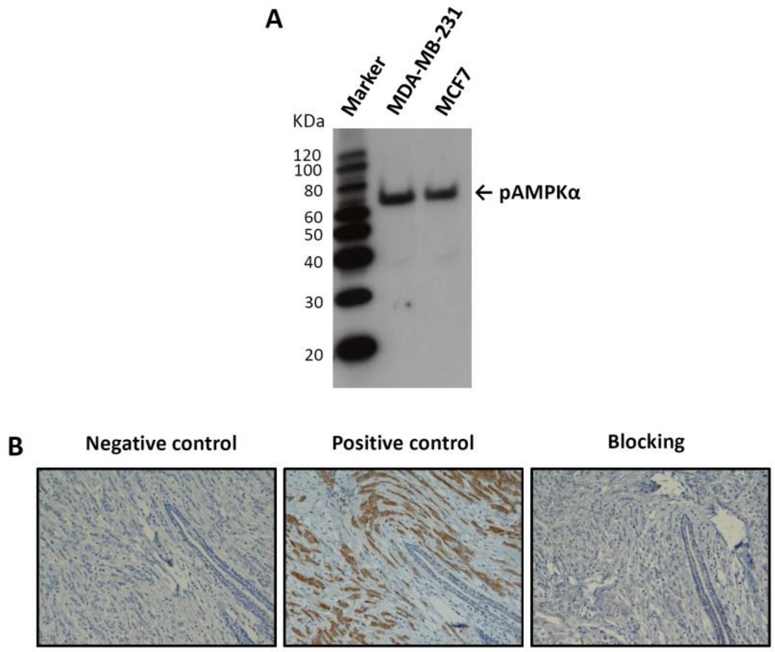


**Figure S1:** Antibody specificity validation. (A) Lysates from human breast cancer MDA-MB-231 and MCF7 cells were analyzed by Western blot to assess expression of pAMPKα. (B) A peptide blocking assay was conducted to confirm the immunohistochemical staining specificity. Breast tumor sections were subjected to IHC without antibody (negative control), with anti-AMPKα antibody (positive control), or with the anti-AMPKα antibody pre-incubated with AMPKα1 + AMPKα2 peptide (blocking). The immunohistochemical staining pattern for each section is shown using photomicrographs at x20 microscopic magnification.
